# Health-related quality of life and income-related social mobility in young adults

**DOI:** 10.1186/1477-7525-12-52

**Published:** 2014-04-15

**Authors:** David S Brennan, A John Spencer

**Affiliations:** 1 Australian Research Centre for Population Oral Health School of Dentistry, University of Adelaide, Adelaide 5005, South Australia

**Keywords:** Life-course, Mobility, Young adults, OHIP, EQ-VAS, SWLS

## Abstract

**Background:**

To assess the association of income-related social mobility between the age of 13 and 30 years on health-related quality of life among young adults.

**Methods:**

In 1988-89 n = 7,673 South Australian school children aged 13 years were sampled with n = 4,604 children (60.0%) and n = 4,476 parents (58.3%) returning questionnaires. In 2005-06 n = 632 baseline study participants responded (43.0% of those traced and living in Adelaide).

**Results:**

Multivariate regressions adjusting for sex, tooth brushing and smoking status at age 30 showed that compared to upwardly mobile persons social disadvantage was associated (p < 0.05) with more oral health impact (Coeff = 5.5), lower EQ-VAS health state (Coeff = -5.8), and worse satisfaction with life scores (Coeff = -3.5) at age 30 years, while downward mobility was also associated with lower satisfaction with life scores (Coeff = -1.3).

**Conclusions:**

Stable income-related socioeconomic disadvantage was associated with more oral health impact, and lower health state and life satisfaction, while being downwardly mobile was associated with lower life satisfaction at age 30 years. Persons who were upwardly mobile were similar in health outcomes to stable advantaged persons.

## Background

Exploration of social determinants of health have considered theoretical approaches including life-course analysis [1]. Life-course explanations of health inequalities have looked at the interrelations of materialist, behavioural and psychosocial factors [2], and at how determinants of health across the life-course may affect disease risk [3]. Life-course models of how determinants of health across the life-course may affect disease risk have included critical periods, accumulation of risk, and social mobility [3].

Social mobility models classify change in socioeconomic status (SES) over time into upwardly or downwardly mobile categories as well as stable SES for some people over their life-course [4]. Examples of measures used to define social mobility have included income, occupation, education and social class [5-8]. Cohort studies of oral health have shown socioeconomic trajectories to be related to adult oral health, with the poorest oral health among those with consistently low socioeconomic position during their life-course [9].

The Wilson and Cleary model of health outcomes links physiological variables, symptoms, functional health, general health perceptions and overall quality of life [10,11]. Oral health studies of social mobility can extend their explanatory scope by inclusion of general health and dimensions of well-being as outcomes. Oral health has been associated with general health [12-14], and for older adults those in worse general health reported to suffer more impact from oral health problems [15].

In this study the aim was to assess the association of social mobility between the age of 13 and 30 years on health-related quality of life among young adults using measures of oral health impact, general health state and well-being. The study therefore adds to the evidence base of life course models of oral health by broadening the outcomes being assessed. Specifically, it includes oral health impact, general health and well-being. In doing so, it sheds light on a range of levels from the Wilson and Cleary model from function, to general health perceptions and overall quality of life. Further, the measurement of social mobility contrasts upward mobility to assess the potential positive effect of such social mobility with those in stable or downward mobility groups.

## Methods

### Sampling and data collection

Subjects for the study were drawn from students enrolled in the South Australian School Dental Service (SDS). All 13 year-old students due for a recall appointment at the SDS clinics over the sampling period from November 1988 to July 1989 were invited to participate in the study. The sample comprised n = 7,673 13 year-old children attending for recall examinations at the SDS during 1988-1989, with n = 4,476 parents giving consent. Data were collected from parents and children by mailed survey. SDS staff collected oral health and dental treatment data. Of the original sample 66% were from metropolitan Adelaide. Sample contact details were updated in 2005-06 using the Electoral Roll. Those residing in metropolitan Adelaide aged around 30 years old (comprising n = 1,859 or 41.5% of the original 4,476 consenting) were surveyed by mailed questionnaire including multiple follow-up mailings [16], with questionnaire respondents invited to a dental examination. A random sample of n = 547 similarly aged persons was drawn from the Electoral Roll in 2005-06 as a comparison group to provide an independent population sample to assess the representativeness of the participants followed up in the main study. Ethics clearance was obtained from the Human Research Ethics Committee, University of Adelaide.

### Outcomes

Oral health-related impact was measured using the OHIP-14 from the questionnaire at age 30 years in 2005-06 [17]. OHIP-14 has good reliability, validity and precision [17], and associations with explanatory variables were not substantially diminished by affectivity [18]. The OHIP-14 uses 14 items to capture measures of the seven dimensions of functional limitation (comprising items relating to ‘trouble pronouncing words’ and ‘sense of taste worsened’), physical pain (with items ‘painful aching in mouth’ and ‘uncomfortable eating’), psychological discomfort (with items ‘self-conscious’ and ‘tense’), physical disability (with items ‘diet unsatisfactory’ and ‘interrupt meals’), psychological disability (with items ‘difficulty relaxing’ and ‘embarrassed’), social disability (with items ‘irritable’ and ‘difficulty with usual jobs’) and handicap (with items ‘life less satisfying’ and ‘unable to function’). For each item participants rate the frequency of impacts in the preceding 12 months coded as 4 = very often, 3 = fairly often, 2 = occasionally, 1 = hardly ever and 0 = never which are summed to produce an OHIP score that could range from 0 to 56. Higher scores indicate greater impact of dental problems.

General health-related quality of life was collected using the visual analogue scale of the EuroQol, a standardised generic instrument for describing and valuing health-related quality of life [19]. This was performed by placing a mark on a thermometer-like scale (EQ-VAS) ranging from zero (worst possible health) to 100 (best possible health). Higher EQ-VAS scores indicate better general health.

Well-being was measured using the Satisfaction With Life Scale (SWLS), comprising five-items measured on a 5-point Likert scale where 1 = strongly disagree, 2 = disagree, 3 = neutral, 4 = agree and 5 = strongly agree [20], with the scale score created by summing the responses to the items. Higher SWLS scores indicate a higher level of well-being.

### Explanatory variables

Social mobility was assessed using family income when the participant was aged 13 and own income at age 30 years. At age 13 the parents or guardians were asked to provide the weekly total family income (before tax) in one of five income categories. At age 30 the study participants were asked to provide the total (gross or before-tax) yearly income of their household in one of nine categories. To allow for the different income categories used and difference in levels of income between the two points of data collection at age 13 and age 30 a distributional approach to classifying income was adopted. Income was classified into approximate tertiles. Those in the same income tertile at both points in time were classified as ‘disadvantaged’ for those in the lower income tertiles, ‘middle’ for those in the middle income tertiles, and ‘advantaged’ for those in the high income tertiles. Those who moved from middle to lower tertiles, or from upper to middle or to lower tertiles, were classified as ‘downwardly mobile’. Those who moved from middle to upper tertiles, or from lower to middle or to upper tertiles, were classified as ‘upwardly mobile’.

Covariates of sex, tooth brushing frequency and smoking status were collected in the questionnaire at age 30 years. Tooth brushing was coded as ‘more than once a day’ and ‘once a day or less’. Smoking was recorded between the age of 13 and 30 years, and classified as current or former smokers, and those that never smoked. Tooth brushing and smoking were considered as covariates due to the potential influence of such health behaviours on the outcomes. These could include direct effects as well as potentially operating as markers for other unmeasured variables not captured directly in the covariates. Covariates were included to control for potential confounding rather than to model their effects. Hence, dichotomised variables were included as a parsimonious representation of their effects while providing sufficient numbers of people in each subgroup.

### Analysis

Response rates were adjusted for subjects that could not be contacted (i.e., not residing at the traced address). Bias was examined by comparing baseline characteristics of respondents in 2005-06 versus non-respondents, and by comparing the characteristics of baseline participants who responded in 2005-06 versus the comparison group who responded in 2005-06 using t-tests and chi-square. Bivariate associations of the outcomes by explanatory variables were tested using Ordinary Least Squares (OLS) regression, with regression coefficients from multivariable models reported as adjusted effects. Covariates were retained in the adjusted models regardless of their level of statistical significance in the bivariate analysis. The upwardly mobile group was adopted as the reference category for income-related SES mobility, reflecting the aim of assessing the potential positive effect of such mobility with those in stable or downward groups. OLS regression was performed, reflecting the continuous nature of the distribution of the outcome variable scores.

## Results

### Response

In 1988-89 n = 4,604 children (60.0%) and n = 4,476 parents (58.3%) returned questionnaires, with 3,925 students (51.1%) examined by the SDS and both parents and students completed questionnaires. In 2005-06 n = 632 baseline participants responded (43.0% of those traced and living in Adelaide or 14.1% of the original 4,476 giving consent in 1988-89), with n = 145 persons from the newly sampled comparison group (33.9% response).

### Comparison of baseline characteristics

Respondents in 2005-06 were compared with non-respondents using data from their baseline characteristics collected in 1988-89. Respondents in 2005-06 had higher percentages female, with tertiary educated parents and with male parents working, but lower percentages covered by a health care card and from higher household income groups. There were no differences between respondents in 2005-06 and non-respondents in the baseline characteristics of country of birth or occupation of either parent or employment status of female parents There were also no differences in oral health at age 13 years (i.e., oral hygiene, calculus, DAI score, number of permanent teeth that were restored sound, carious, unsatisfactory restorations, needing extraction, extracted due to caries and DMFT score).

### Similarity to newly sampled comparison group

Comparison of baseline participants who responded in 2005-06 and the similar age comparison group sampled in 2005-06 showed the longitudinal respondents (i.e., with data from 1988-89 and 2005-06) were more likely to be working. The longitudinal respondents were no different to the newly sampled comparison group in sex, country of birth, education level, income, or health card status. They were also no different in time since last visit, reason for last visit and toothbrushing frequency. However, they had a higher rate of fillings in the past year than the comparison group but were not different on number of visits, examinations, scale and clean services, and extractions. The longitudinal respondents were not different to the comparison group in numbers of decayed, missing and filled teeth.

### Distributions and unadjusted associations

The outcome variables were distributed as follows: OHIP (mean = 6.1, Std Dev = 7.8, min = 0.0, max = 46.0, median = 4.0, interquartile range = 8.0), EQ-VAS (mean = 80.0, Std Dev = 13.0, min = 30.0, max = 100.0, median = 80.0, interquartile range = 15.0), SWLS (mean = 18.4, Std Dev = 4.0, min = 5.0, max = 25.0, median = 19.0, interquartile range = 5.0). A fifth were classified as advantaged (20.9%), 22.9% as upwardly mobile, 10.5% as stable middle income, 37.5% as downwardly mobile and 8.3% as disadvantaged (Table 1). While not statistically significant the stable disadvantaged group tended to have a lower percentage of males and the lowest percentage brushing their teeth more than once a day. Smoking varied significantly across the income-related SES mobility groups, being lowest among the stable advantaged group.

**Table 1:** **Distribution of income**-**related SES mobility**, **and associations with covariates at age 30**

	**Distribution**		**Age 30 characteristics**		
	**N**	**%**	**% males**	**% brushing > 1 per day**	**% cigarette smokers**
**Income-related SES mobility**			NS	NS	**
Stable advantaged	86	20.9	46.5	79.3	22.1
Upwardly mobile	94	22.9	48.9	72.8	33.0
Stable middle	43	10.5	55.8	62.8	51.2
Downwardly mobile	154	37.5	42.2	67.1	42.2
Stable disadvantaged	34	8.3	29.4	58.8	41.2

*(P < 0.05), **(P < 0.01).

In unadjusted analyses both OHIP and SWLS were associated with income-related SES mobility, with the stable disadvantaged group having the highest level of oral health impact and the lowest level of satisfaction with life (Table 2). While not reaching statistical significance, the lowest EQ-VAS scores occurred in the stable disadvantaged group.

**Table 2:** **Unadjusted associations with health**-**related quality of life variables at age 30**

	**OHIP**-**14**		**EQ**-**VAS**		**SWLS**	
	**Mean**	**(SE)**	**Mean**	**(SE)**	**Mean**	**(SE)**
**Income**-**related SES mobility**	**		NS		**	
Stable advantaged	4.3	(0.5)	79.8	(1.5)	19.5	(0.4)
Upwardly mobile	5.6	(0.6)	82.3	(1.2)	19.4	(0.3)
Stable middle	5.8	(0.9)	81.4	(1.8)	18.1	(0.6)
Downwardly mobile	5.8	(0.6)	78.8	(1.1)	18.0	(0.3)
Stable disadvantaged	11.6	(1.8)	75.8	(2.6)	15.8	(0.7)
**Sex**	NS		NS		NS	
Male	5.4	(0.4)	79.8	(0.9)	18.0	(0.3)
Female	6.6	(0.5)	80.2	(0.8)	18.6	(0.2)
**Tooth brushing frequency**	*		**		*	
Once a day or more	5.6	(0.4)	81.3	(0.7)	18.6	(0.2)
Less than once per day	7.4	(0.7)	77.0	(1.3)	17.7	(0.4)
**Cigarette smoking**	**		NS		**	
Non-smoker	5.0	(0.3)	80.6	(0.8)	18.8	(0.2)
Current/former smoker	7.8	(0.7)	79.0	(1.0)	17.7	(0.3)
**All persons**	6.1	(0.3)	80.0	(0.6)	18.4	(0.2)

*(P < 0.05), **(P < 0.01).

There were no sex differences in OHIP, EQ-VAS or SWLS scores. Less frequent tooth brushing was associated with higher OHIP scores and lower scores for EQ-VAS and SWLS. Smoking was associated with higher OHIP scores and lower SWLS scores.

### Multivariable models

The adjusted models showed that income-related SES mobility was related to OHIP, EQ-VAS and SWLS scores (Table 3). Compared to the reference category of upwardly mobile persons those in the stable social disadvantage group had (p < 0.05) more oral health impact (Coeff = 5.5), lower EQ-VAS (Coeff = -5.8), and worse satisfaction with life scores (Coeff = -3.5) at age 30 years, while downward mobility was also associated with lower satisfaction with life scores (Coeff = -1.3). More frequent tooth brushing was associated with less oral health impact as indicated by lower OHIP scores (Coeff = -1.5) and higher health state as indicated by higher EQ-VAS ratings (Coeff = 4.2). Higher oral health impact was associated with smoking as indicated by higher OHIP scores (Coeff = 2.4).

**Table 3:** **Adjusted associations with health**-**related quality of life variables at age 30**

	**OHIP**-**14**			**EQ**-**VAS**			**SWLS**		
	**Coeff.**	**(SE)**	**P**	**Coeff.**	**(SE)**	**P**	**Coeff.**	**(SE)**	**P**
**Income**-**related SES mobility**									
Stable advantaged	-1.0	(1.0)	NS	-3.0	(2.0)	NS	0.1	(0.6)	NS
Upwardly mobile (Ref.)	-	(-)		-	(-)		-	(-)	
Stable middle	-0.5	(1.2)	NS	-0.4	(2.4)	NS	-1.0	(0.7)	NS
Downwardly mobile	-0.2	(0.9)	NS	-3.2	(1.7)	NS	-1.3	(0.5)	*
Stable disadvantaged	5.5	(1.3)	**	-5.8	(2.6)	*	-3.5	(0.8)	**
**Sex**									
Male	-0.6	(0.7)	NS	-0.0	(1.3)	NS	-0.6	(0.4)	NS
Female (Ref.)	-	(-)		-	(-)		-	(-)	
**Tooth brushing frequency**									
Once a day or more	-1.5	(0.7)	*	4.2	(1.4)	**	0.5	(0.4)	NS
Less than once per day (Ref.)	-	(-)		-	(-)		-	(-)	
**Cigarette smoking**									
Non-smoker (Ref.)	-	(-)		-	(-)		-	(-)	
Current/former smoker	2.4	(0.7)	**	-0.9	(1.4)	NS	-0.6	(0.4)	NS

*(P < 0.05), **(P < 0.01).

## Discussion

This study showed that SES disadvantage as measured by income was associated with more oral health impact, worse general health and lower satisfaction with life. The generally worse condition of those with a stable level of income-related disadvantage suggests a cumulative impact of oral health problems over time. In addition, being downwardly mobile was also associated with lower satisfaction with life. However, the contrasting patterns of those who were downwardly mobile compared to those who were upwardly mobile suggests proximate factors around age 30 may be important [5]. This may be reflect young adulthood being a life-stage of transition and growing independence [21].

Oral health impact and general health were also associated with more frequent tooth brushing, while oral health impact was also associated with smoking. This could reflect direct effects or clustering of preventive or risky health behaviours [22]. EQ-5D has been associated with oral health factors [12,23]. Tooth brushing has been associated with dental caries [24,25], most likely reflecting exposure to fluoridated toothpaste, and smoking associated with periodontal disease [26,27].

Baseline participants were representative of demographic variables of the population [28]. Loss to follow-up was from lower socio-economic groups, but there was no difference in baseline oral health. However, differences in baseline socioeconomic status could impact on oral health outcomes as the study participants moved from childhood to adulthood [29]. Imputation of data was not attempted as the issue was loss to follow-up of cases rather than missing data items. Statistical power was considered adequate as the models reached statistical significance for differences in all three outcomes by the income-related mobility groups. Hence, bias analysis was conducted which showed loss to follow-up from low SES groups but no differences in oral health at baseline. Comparison with a similarly aged group from 2005-06 showed the longitudinal respondents were more likely to be working, but not different in caries experience. So, loss to follow-up from lower socio-economic groups is a limitation of the study. The follow-up period spanning a meaningful transition or life stage progression from childhood to young adulthood that incorporated a range of outcome measures would be considered as strengths.

In this study we examined the life-course model of social mobility based on income. Other reports of life-course models of adult oral health include those from the UK using social class to measure SES [6], Brazil using income to define SES [7], New Zealand using occupation as a marker of SES [8], and Finland using education as an SES measure [5]. The findings from New Zealand and Brazil have highlighted SES mobility in relation to oral health of young adults [9], consistent with the present study. There is evidence to support other life-course approaches such as critical period and accumulation of risk as well as social mobility models. However, these life-course models are not strictly separable as competing hypotheses and may operate together [30]. Interpretation of their effects requires prior knowledge regarding causal mechanisms as there is no critical test to disentangle the mutual confounding of the three life-course models. However, nested models of alternative life course approaches can be assessed against an all-inclusive saturated model [31], and may complement other analytical approaches in life course epidemiology with the caveat that complex life course trajectories may be difficult to represent in simple statistical models [32].

The associations of income-related social mobility with measures such as EQ-VAS and SWLS indicate the value of broadening oral health cohort studies through the inclusion of general health state measures, and the incorporation of higher level quality of life measures that tap into well-being such as satisfaction with life. Oral health impact, while lowest in the stable advantaged group and highest in the stable disadvantaged group, was also generally lower in all groups compared with the stable disadvantaged. For general health, the EQ-VAS scores were lowest in the stable disadvantaged group but highest in the upwardly mobile group. The pattern by income-related social mobility for well-being tended to be more of a gradient, from higher levels in the stable advantaged and upwardly mobile groups, through to lower levels in the stable middle and downwardly mobile groups, and lowest in the stable disadvantaged group.

The findings underscore the importance of social circumstances to health across a range of outcome levels that include impact of oral health problems, general health state, and well-being. Further, it has been reported that SES modified the relationship between child oral health and dental caries at age 30 years [33], with those who were lower SES in childhood being worse off in oral health at age 30 when controlling for differences in childhood caries. This may support social causation explanations for oral health inequalities where SES gradients in health reflect the impact of SES on health through such mechanisms as career prospects and access to goods and services [34]. Education attainment may also be another important factor related to health through the life course. Parental education may contribute to adult oral health during a critical period, while education may also have a cumulative effect during the life course as well as having a proximal effect in terms of mobility and adult oral health [5]. Thus policy options may be developed to address health inequalities at various life stages [1], and to tackle upstream issues that underlie the social determinants of health [35].

## Conclusions

Stable income-related socioeconomic disadvantage was associated with more oral health impact, and lower health state and life satisfaction, while being downwardly mobile was associated with lower life satisfaction at age 30 years. Upwardly mobile persons were similar in health outcomes to stable advantaged persons.

## Abbreviations

EQ-VAS: EuroQol visual analogue scale; DAI: Dental Aesthetic Index; DMFT: Decayed, missing and filled teeth; OHIP-14: Oral Health Impact Profile 14-item version; OLS: Ordinary Least Squares; SDS: School Dental Service; SES: Socioeconomic status; SWLS: Satisfaction With Life Scale.

## Competing interests

The authors declare that they have no competing interests.

## Authors’ contributions

DSB performed analyses and drafted the manuscript. AJS was involved in developing the project and revising the manuscript. Both authors have read and approved the manuscript.
